# Pregnancy and perinatal outcomes following PIEZO-ICSI with vitrified-warmed single blastocyst transfer: a retrospective cohort study

**DOI:** 10.1007/s10815-026-03915-0

**Published:** 2026-05-23

**Authors:** Kenichiro Hiraoka, Miki Osugi, Moe Sato, Yuko Kamada, Hayata Nakajo, Toshio Sujino, Akira Komiya, Yurie Nako, Yuko Takayanagi, Makiko Tajima, Tatsuyuki Ogawa, Kumi Ohuchi, Masaru Hayashi, Kiyotaka Kawai

**Affiliations:** 1 Kameda IVF Clinic Makuhari, Chiba, Japan; 2 Institute of SCIENCE TOKYO, Tokyo, Japan; 3 Kameda Medical Center, Kamogawa, Japan; 4 Omotesando ART Clinic, Tokyo, Japan

**Keywords:** Humans, In vitro fertilization (IVF), Perinatal outcomes, PIEZO-ICSI, Sex ratio

## Abstract

**Objective:**

To report pregnancy and perinatal outcomes following intracytoplasmic sperm injection using a piezo element (PIEZO-ICSI) in a large Japanese cohort undergoing vitrified-warmed single blastocyst transfer. Outcomes from conventional IVF cycles were included as a reference population sharing similar ovarian stimulation and embryo transfer protocols.

**Methods:**

This study was a retrospective cohort study conducted in two fertility centers in Japan. A total of 1,822 PIEZO-ICSI cycles from 1,072 patients aged < 40 years at oocyte retrieval were included between June 2016 and August 2023. A reference population of 1,701 IVF cycles from 996 patients was included for context.

**Results:**

In the PIEZO-ICSI cohort, the clinical pregnancy rate was 49.9% (910/1,822) and the live birth rate was 38.7% (706/1,822). Among live births, the mean birth weight was 3,011.4 ± 467.9 g, mean gestational age was 38.59 ± 1.95 weeks, and congenital anomaly rate was 1.6% (11/706). These outcomes were comparable to the IVF reference population (clinical pregnancy rate 48.4%, live birth rate 37.9%, birth weight 3,022.8 ± 469.5 g, congenital anomaly rate 2.5%). The proportion of female infants was significantly higher in the PIEZO-ICSI group (53.2% vs. 43.4%; *P* < 0.001).

**Conclusion:**

PIEZO-ICSI is associated with favorable pregnancy and perinatal outcomes in women aged < 40 years.

**Supplementary Information:**

The online version contains supplementary material available at 10.1007/s10815-026-03915-0.

## Introduction

Intracytoplasmic sperm injection (ICSI) has become a cornerstone of assisted reproductive technology (ART) since its introduction in 1992 [1]. Standard ICSI involves the use of a micropipette with a beveled and spiked tip to penetrate the zona pellucida and aspirate the cytoplasm to induce membrane rupture. In contrast, PIEZO-ICSI utilizes a flat-tipped micropipette and applies an ultra-fast submicron-level suction generated by a piezo pulse to open the zona pellucida and disrupt the membrane without aspirating cytoplasm into the micropipette [2].

Several studies have demonstrated favorable fertilization and embryo development outcomes with PIEZO-ICSI compared with standard ICSI [3–11]. PIEZO-ICSI has shown higher fertilization rates and improved blastocyst development, particularly in women over 35 years of age and in patients with poor prognosis [6, 10]. A recent multicenter sibling oocyte study confirmed improved fertilization rates, lower degeneration rates, and higher good-quality embryo rates with PIEZO-ICSI compared to conventional ICSI [11].

Despite these encouraging laboratory outcomes, the safety profile of PIEZO-ICSI regarding pregnancy and perinatal outcomes remains uncertain due to limited available data [12]. While numerous studies have examined perinatal outcomes following standard ICSI compared with conventional IVF [13–21], data specifically on PIEZO-ICSI outcomes are scarce. Given the increasing adoption of PIEZO-ICSI worldwide, establishing its safety profile is of paramount importance.

The objective of this study was to report pregnancy and perinatal outcomes following PIEZO-ICSI in a large cohort of Japanese women undergoing vitrified-warmed single blastocyst transfer. To provide clinical context, outcomes from conventional IVF cycles performed at the same institutions during the same period were included as a reference population. IVF serves as an appropriate reference because these cycles share similar ovarian stimulation protocols, laboratory culture conditions, vitrification techniques, and embryo transfer procedures, differing only in the fertilization method. Multiple sensitivity analyses were performed to enhance the robustness of our findings.

## Materials and methods

### Study design and population

This retrospective cohort study included patients who underwent vitrified-warmed embryo transfer at Kameda Medical Center and Kameda IVF Clinic Makuhari between June 2016 and August 2023. All embryos, without preimplantation genetic testing (PGT), were derived from fresh, non-donor oocytes fertilized using either conventional IVF or PIEZO-ICSI with ejaculated spermatozoa. During the study period, only PIEZO-ICSI was exclusively performed at both clinics; standard ICSI was not available.

The choice between IVF and PIEZO-ICSI was determined by the following criteria: PIEZO-ICSI was indicated for couples with male factor infertility (oligozoospermia, asthenozoospermia, teratozoospermia, or combinations thereof), previous fertilization failure with IVF, or patient preference. Conventional IVF was performed for couples without male factor infertility when semen parameters were within normal ranges according to WHO 5th edition criteria.

Although conventional IVF was generally indicated for couples without male factor infertility, some patients classified as having male factor infertility based on the initial semen analysis underwent IVF. This occurred when semen parameters on the oocyte retrieval day showed improvement and met WHO 5th edition reference values for IVF. The couple preferred to proceed with IVF after counseling.

Inclusion criteria were: (1) vitrified-warmed single blastocyst transfer cycles, (2) female age < 40 years at oocyte retrieval, (3) Day 5 or Day 6 blastocyst transfer, and (4) frozen embryo transfer (natural or hormone replacement cycle). The age restriction of < 40 years was applied to minimize the confounding effects of advanced maternal age on pregnancy and perinatal outcomes, including increased risks of chromosomal abnormalities, miscarriage, and obstetric complications, thereby allowing more precise evaluation of the fertilization technique itself. Exclusion criteria were: (1) fresh embryo transfer cycles, (2) sequential transfer cycles, (3) double embryo transfer cycles, and (4) cycles involving 1-day-old PIEZO-ICSI embryos.

### Clinical and laboratory protocols

Ovarian stimulation was performed using either a gonadotropin-releasing hormone (GnRH) agonist long protocol or a GnRH antagonist protocol, as previously described [22]. Oocyte retrieval was performed 35–36 h after human chorionic gonadotropin (hCG) or GnRH agonist trigger.

For conventional IVF, oocytes were inseminated 4–6 h after retrieval with approximately 16,000 motile spermatozoa per oocyte. For PIEZO-ICSI, cumulus cells were removed 2–3 h after retrieval, and ICSI was performed using a piezo drive unit (PMM-150FU; Prime Tech, Ibaraki, Japan) with a flat-tipped micropipette (inner diameter 6 μm, wall thickness 0.625 μm), as previously described [5, 8]. The operational liquid was Fluorinert FC-77 (3M, St. Paul, MN, USA), which transmits the piezo pulse to the micropipette.

Fertilization was assessed 16–18 h after insemination by the presence of two pronuclei. Normally fertilized oocytes were cultured in a single-step culture medium (ONESTEP Medium, NAKA Medical Inc., Japan) until the blastocyst stage. Blastocyst grading was performed according to Gardner’s criteria [23].

Only expanded blastocysts (Gardner grade ≥ 4) were cryopreserved using the Cryotop vitrification method with commercially available vitrification and warming media (Oocyte/Embryo Vitrification and Thawing Media, Kitazato Corporation, Japan). Assisted hatching was performed on all warmed blastocysts by opening approximately 50% of the zona pellucida using a diode laser [24]. Warmed blastocysts were transferred onto a Day 5 endometrium in either a natural cycle with or without hCG trigger, or a hormone replacement cycle using estradiol valerate and progesterone.

### Outcome measures

The primary outcomes were clinical pregnancy rate and live birth rate. Clinical pregnancy was defined as the presence of a gestational sac with fetal heartbeat confirmed by transvaginal ultrasonography at 7–8 weeks of gestation. Live birth was defined as delivery of a live infant at ≥ 22 weeks of gestation.

Secondary outcomes included: (1) biochemical pregnancy rate (positive serum hCG without subsequent ultrasound evidence of pregnancy), (2) miscarriage rate (spontaneous loss of clinical pregnancy before 22 weeks of gestation), (3) ectopic pregnancy rate, (4) cesarean section rate, (5) pregnancy complications (preterm delivery < 37 weeks, hypertensive disorders of pregnancy, gestational diabetes mellitus, placenta previa, placental abruption), (6) birth weight, (7) gestational age at delivery, (8) infant sex, and (9) congenital anomalies diagnosed at birth or within the first month after birth.

### Statistical analysis

Continuous variables are presented as mean ± standard deviation (SD) and were compared using the Mann–Whitney U test because of non-normal distributions. Categorical variables are presented as frequencies and percentages and were compared using Fisher's exact test.

The primary analysis presents outcomes of the PIEZO-ICSI cohort, with the IVF cohort serving as a reference population for clinical context. To assess the consistency and robustness of the findings, the following sensitivity analyses were performed:

1. First frozen embryo transfer (FET) only analysis: To address the potential bias from including multiple cycles per patient, we restricted the analysis to each patient's first FET cycle during the study period.
2. Propensity score matching (PSM): To balance baseline characteristics between groups, propensity scores representing the probability of receiving PIEZO-ICSI were calculated using logistic regression with maternal age at oocyte retrieval and age at embryo transfer as covariates. One-to-one nearest neighbor matching without replacement was performed using a caliper width of 0.2 times the standard deviation of the logit of the propensity score.
3. Stratified analysis: To assess whether outcomes differed by indication, we performed stratified analysis by the presence or absence of male factor infertility.
4. Multivariable logistic regression: To estimate the adjusted effect of fertilization method on live birth while controlling for potential confounders, we performed multivariable logistic regression with the following covariates: fertilization method (PIEZO-ICSI vs. IVF), maternal age at oocyte retrieval, transfer cycle number, Day 6 embryo (vs. Day 5), and hormone replacement cycle (vs. natural cycle). Results are presented as adjusted odds ratios (aOR) with 95% confidence intervals (CI) calculated using bootstrap resampling (1,000 iterations).

All statistical tests were two-sided, and *P* < 0.05 was considered statistically significant. Statistical analyses were performed using Python version 3.10 with SciPy version 1.11 and scikit-learn version 1.3 libraries.

## Results

### Study population

A total of 4,135 vitrified-warmed embryo transfer cycles were initially screened. After applying inclusion and exclusion criteria, 3,523 cycles from 2,068 patients were included in the analysis. The PIEZO-ICSI cohort comprised 1,822 cycles from 1,072 patients, and the IVF reference population comprised 1,701 cycles from 996 patients.

Patient characteristics are shown in Table 1. The PIEZO-ICSI and IVF groups were comparable in terms of maternal age at oocyte retrieval (34.4 ± 3.5 years vs. 34.4 ± 3.5 years; *P* = 0.82), body mass index (21.5 ± 3.3 kg/m^2^ vs. 21.5 ± 3.2 kg/m^2^; *P* = 0.76), anti-Müllerian hormone levels (2.9 ± 2.8 ng/mL vs. 2.9 ± 2.7 ng/mL; *P* = 0.55), and number of oocytes retrieved (12.0 ± 6.9 vs. 12.5 ± 7.9; *P* = 0.51). The number of transfer cycles differed statistically between the groups (1.8 ± 1.2 vs. 1.7 ± 1.0, *P* < 0.001), although the absolute difference was small. As expected given the indications for each technique, the proportion of male factor infertility was higher in the PIEZO-ICSI group (68.4% vs. 33.9%; *P* < 0.001).

**Table 1:** Patient and cycle characteristics

Characteristic	PIEZO-ICSI (*n* = 1,822)	IVF reference (*n* = 1,701)	*P* value
Number of patients	1,072	996	
Maternal age at retrieval (years)	34.4 ± 3.5	34.4 ± 3.5	0.82
Maternal age at transfer (years)	35.0 ± 3.6	35.0 ± 3.5	0.75
Male partner age (years)	37.7 ± 6.1	36.7 ± 4.9	< 0.001
BMI (kg/m^2^)	21.5 ± 3.3	21.5 ± 3.2	0.76
AMH (ng/mL)	2.9 ± 2.8	2.9 ± 2.7	0.55
Number of oocytes retrieved	12.0 ± 6.9	12.5 ± 7.9	0.51
Number of mature oocytes	9.7 ± 5.7	10.4 ± 6.5	0.02
Transfer cycle number	1.8 ± 1.2	1.7 ± 1.0	< 0.001
Infertility etiology			
Male factor	1,246 (68.4%)	577 (33.9%)	< 0.001
Tubal factor	210 (11.5%)	299 (17.6%)	< 0.001
Unexplained	320 (17.6%)	575 (33.8%)	< 0.001

Data are presented as mean ± SD or *n* (%). *BMI* body mass index, *AMH* anti-Müllerian hormone

### Pregnancy outcomes following PIEZO-ICSI

Pregnancy outcomes are summarized in Table 2. In the PIEZO-ICSI cohort, the biochemical pregnancy rate was 59.8% (1,089/1,822), the clinical pregnancy rate was 49.9% (910/1,822), and the live birth rate was 38.7% (706/1,822). Among clinical pregnancies, the miscarriage rate was 20.0% (182/910).

**Table 2:** Pregnancy outcomes

Outcome	PIEZO-ICSI (*n* = 1,822)	IVF reference (*n* = 1,701)	*P* value
Biochemical pregnancy rate	1,089/1,822 (59.8%)	1,019/1,701 (59.9%)	0.95
Clinical pregnancy rate	910/1,822 (49.9%)	824/1,701 (48.4%)	0.38
Live birth rate	706/1,822 (38.7%)	644/1,701 (37.9%)	0.60
Miscarriage rate*	182/910 (20.0%)	156/824 (18.9%)	0.59
Ectopic pregnancy rate	8/1,822 (0.4%)	13/1,701 (0.8%)	0.19

*Calculated among clinical pregnancies

These outcomes were comparable to the IVF reference population: biochemical pregnancy rate 59.9% (1,019/1,701; *P* = 0.95), clinical pregnancy rate 48.4% (824/1,701; *P* = 0.38), live birth rate 37.9% (644/1,701; *P* = 0.60), and miscarriage rate 18.9% (156/824; *P* = 0.59).

### Perinatal outcomes following PIEZO-ICSI

Perinatal outcomes are shown in Table 3. Among 706 live births following PIEZO-ICSI, the cesarean section rate was 35.0% (247/706), the pregnancy complication rate was 23.8% (168/706), the preterm delivery rate (< 37 weeks) was 8.8% (62/706), the mean birth weight was 3,011.4 ± 467.9 g, and the mean gestational age at delivery was 38.59 ± 1.95 weeks. The congenital anomaly rate was 1.6% (11/706).

**Table 3:** Perinatal outcomes among live births

Outcome	PIEZO-ICSI (*n* = 706)	IVF reference (*n* = 644)	*P* value
Cesarean section	247/706 (35.0%)	224/644 (34.8%)	0.95
Pregnancy complications	168/706 (23.8%)	166/644 (25.8%)	0.41
Preterm delivery (< 37 weeks)	62/706 (8.8%)	65/644 (10.1%)	0.43
Birth weight (g)	3,011.4 ± 467.9	3,022.8 ± 469.5	0.39
Gestational age (weeks)	38.59 ± 1.95	38.61 ± 2.09	0.45
Infant sex			
Male	325/695 (46.8%)	363/641 (56.6%)	< 0.001
Female	370/695 (53.2%)	278/641 (43.4%)	< 0.001
Congenital anomalies	11/706 (1.6%)	16/644 (2.5%)	0.25

Data are presented as mean ± SD or *n* (%)

These outcomes were comparable to the IVF reference population: cesarean section rate 34.8% (*P* = 0.95), pregnancy complication rate 25.8% (*P* = 0.41), preterm delivery rate 10.1% (*P* = 0.43), birth weight 3,022.8 ± 469.5 g (*P* = 0.39), gestational age 38.61 ± 2.09 weeks (*P* = 0.45), and congenital anomaly rate 2.5% (*P* = 0.25). The types of congenital anomalies observed in both groups included cardiac, limb, renal/urinary, gastrointestinal, and chromosomal abnormalities, with no apparent concentration of a specific anomaly type in either group. A complete list of congenital anomalies is provided in Supplementary Table 1.

Notably, the proportion of female infants was significantly higher in the PIEZO-ICSI group compared with the IVF reference population (53.2% [370/695] vs. 43.4% [278/641]; *P* < 0.001).

### Sensitivity analysis 1: First FET only

Analysis restricted to each patient's first FET cycle included 930 PIEZO-ICSI cycles and 971 IVF cycles (Table 4). The clinical pregnancy rate (55.4% vs. 53.1%; *P* = 0.33) and live birth rate (44.7% vs. 41.1%; *P* = 0.12) in the PIEZO-ICSI group were comparable to the IVF reference. The higher proportion of female infants in the PIEZO-ICSI group persisted (52.9% vs. 41.7%; *P* = 0.002).

**Table 4:** Sensitivity analysis: First frozen embryo transfer only

Outcome	PIEZO-ICSI (*n* = 930)	IVF reference (*n* = 971)	*P* value
Number of patients	826	871	
Maternal age at retrieval (years)	33.88 ± 3.57	33.98 ± 3.54	0.58
Clinical pregnancy rate	515/930 (55.4%)	516/971 (53.1%)	0.33
Live birth rate	416/930 (44.7%)	399/971 (41.1%)	0.12
Birth weight (g)*	3,014.2 ± 472.8	3,039.4 ± 488.3	0.25
Female infant*	217/410 (52.9%)	166/398 (41.7%)	0.002

*Among live births

### Sensitivity analysis 2: Propensity score matching

After propensity score matching based on maternal age, 1,700 matched pairs were obtained with excellent baseline balance (Table 5). The live birth rate remained comparable between PIEZO-ICSI and IVF groups (37.1% vs. 37.9%; *P* = 0.65). The difference in sex ratio was consistent, with more female infants in the PIEZO-ICSI group (60.2% vs. 43.4%; *P* < 0.001).

**Table 5:** Sensitivity analysis: Propensity score-matched cohort

Outcome	PIEZO-ICSI (*n* = 1,700)	IVF reference (*n* = 1,700)	*P* value
Maternal age at retrieval (years)	34.35 ± 3.45	34.35 ± 3.45	0.98
Maternal age at transfer (years)	34.92 ± 3.53	34.92 ± 3.54	0.98
Clinical pregnancy rate	904/1,700 (53.2%)	824/1,700 (48.5%)	0.007
Live birth rate	630/1,700 (37.1%)	644/1,700 (37.9%)	0.65
Male infant*	251/630 (39.8%)	363/641 (56.6%)	< 0.001
Female infant*	379/630 (60.2%)	278/641 (43.4%)	< 0.001

*Among live births

### Sensitivity analysis 3: Stratified analysis by male factor status

Stratified analysis demonstrated consistent findings regardless of male factor status (Table 6). In couples with male factor infertility, the live birth rate was 39.6% for PIEZO-ICSI versus 39.2% for IVF (*P* = 0.88). In couples without male factor infertility, the live birth rate was 36.8% for PIEZO-ICSI versus 37.2% for IVF (*P* = 0.92).

**Table 6:** Sensitivity analysis: Stratified by male factor infertility

Outcome	PIEZO-ICSI	IVF reference	P value
With male factor infertility			
Number of cycles	1,246	577	
Clinical pregnancy rate	634/1,246 (50.9%)	285/577 (49.4%)	0.58
Live birth rate	494/1,246 (39.6%)	226/577 (39.2%)	0.88
Without male factor infertility			
Number of cycles	576	1,124	
Clinical pregnancy rate	276/576 (47.9%)	539/1,124 (48.0%)	1.00
Live birth rate	212/576 (36.8%)	418/1,124 (37.2%)	0.92

### Sensitivity analysis 4: Multivariable logistic regression

Multivariable logistic regression was performed to estimate the adjusted effect of fertilization method on live birth (Table 7). After adjusting for maternal age, transfer cycle number, Day 6 embryo, and hormone replacement cycle, the aOR for PIEZO-ICSI versus IVF was 1.056 (95% CI: 0.917–1.224), indicating comparable live birth probability between the two fertilization methods.

**Table 7:** Multivariable logistic regression for live birth

Variable	Adjusted OR	95% CI
PIEZO-ICSI (vs. IVF)	1.056	0.917–1.224
Maternal age at retrieval (per year)	0.905	0.888–0.925
Transfer cycle number (per cycle)	0.852	0.794–0.906
Day 6 embryo (vs. Day 5)	0.514	0.410–0.665
Hormone replacement cycle (vs. natural)	0.892	0.767–1.036

*OR* odds ratio, *CI* confidence interval. Analysis included 3,523 cycles. 95% CI calculated using bootstrap resampling (1,000 iterations). Crude OR for PIEZO-ICSI vs. IVF: 1.038

Other significant predictors of live birth included: maternal age at retrieval (aOR 0.905; 95% CI: 0.888–0.925 per year), transfer cycle number (aOR 0.852; 95% CI: 0.794–0.906 per cycle), and Day 6 embryo (aOR 0.514; 95% CI: 0.410–0.665 vs. Day 5).

## Discussion

This large retrospective cohort study reports favorable pregnancy and perinatal outcomes following PIEZO-ICSI in women aged < 40 years undergoing vitrified-warmed single blastocyst transfer. The live birth rate of 38.7% and congenital anomaly rate of 1.6% are within expected ranges for ART procedures. These outcomes were comparable to a reference IVF population undergoing similar ovarian stimulation and embryo transfer protocols at the same institutions, and this comparability was consistent across multiple sensitivity analyses.

The use of IVF cycles as a reference population, rather than a strict comparator, is appropriate for several reasons. First, both PIEZO-ICSI and IVF cycles in this study underwent identical ovarian stimulation protocols, laboratory culture conditions, vitrification techniques, and embryo transfer procedures. The only difference between groups was the fertilization method. Second, unlike spontaneous conception, both groups experienced the same potential confounders associated with ART, including ovarian hyperstimulation, extended embryo culture, and cryopreservation. Third, given that standard ICSI was not performed at our institutions during the study period, IVF provides the most relevant available comparison within the same clinical and laboratory environment.

The consistency of our findings across multiple analytical approaches strengthens confidence in the results. First FET only analysis addressed the potential bias from including multiple cycles per patient. Propensity score matching balanced baseline characteristics between groups. Stratified analysis by male factor status assessed whether outcomes differed by indication. Multivariable logistic regression quantified the adjusted effect while controlling for potential confounders. All analyses consistently demonstrated comparable live birth rates between PIEZO-ICSI and the IVF reference population.

Although the number of transfer cycles differed statistically between the groups, the absolute difference was minimal (0.1 cycles) and not clinically meaningful. This small difference likely reflects variations in clinical practice and patient preferences rather than differences in prognosis, as major prognostic factors such as maternal age, AMH, BMI, and oocyte yield were well balanced between the groups.

Our finding of a significantly higher proportion of female infants in the PIEZO-ICSI group compared with IVF is consistent with previous reports comparing standard ICSI with IVF [25–27]. This phenomenon has been consistently reported across multiple studies and is thought to be related to differential survival or selection of X-bearing versus Y-bearing spermatozoa during ICSI, or differential embryo survival. The consistency of this finding across all our analytical approaches supports its validity.

Several mechanisms may explain the favorable outcomes observed with PIEZO-ICSI. First, PIEZO-ICSI causes less mechanical stress to the oocyte compared with standard ICSI because membrane rupture is achieved through a controlled piezo pulse without cytoplasmic aspiration, which can damage intracellular structures. Second, the flat-tipped micropipette creates a smaller, more controlled puncture in the zona pellucida and oolemma [2, 5]. Third, the precise control of the piezo pulse allows for consistent and reproducible membrane penetration.

The types of congenital anomalies observed in both groups were heterogeneous and included common categories such as cardiac defects, limb anomalies, and renal or urinary tract anomalies. No specific clustering or pattern of anomalies was observed in the PIEZO-ICSI group. Notably, chromosomal abnormalities (trisomy 21 and trisomy 18) were observed only in the IVF group. These findings support that PIEZO-ICSI does not confer an increased risk of specific congenital anomaly types.

The safety of Fluorinert FC-77, the operational liquid used in PIEZO-ICSI, has been raised as a potential concern. However, our favorable pregnancy and perinatal outcomes, together with previous reports, suggest that Fluorinert does not adversely affect short-term clinical outcomes. At the same time, because our study did not assess long-term offspring health, potential long-term effects cannot be fully excluded. Therefore, long-term follow-up studies are warranted to further evaluate these outcomes. This study has several strengths. It represents one of the largest cohorts reporting pregnancy and perinatal outcomes specifically following PIEZO-ICSI. The use of multiple sensitivity analyses enhances the robustness of our findings. The consistency of results across these approaches provides strong evidence supporting the safety of PIEZO-ICSI.

However, several limitations should be acknowledged. First, the retrospective design may be subject to unmeasured confounders. Second, standard ICSI was not performed at our institutions, precluding direct comparison between PIEZO-ICSI and conventional ICSI. While this limits generalizability, it does not diminish the validity of our outcome reporting for PIEZO-ICSI. Third, the study population was limited to Japanese patients at two centers. Fourth, we restricted analysis to women aged < 40 years to minimize confounding by advanced maternal age; outcomes in older women may differ. Fifth, long-term follow-up data on child health and neurodevelopmental outcomes were not available.

## Conclusions

In conclusion, this large retrospective cohort study demonstrates favorable pregnancy and perinatal outcomes following PIEZO-ICSI in women aged < 40 years undergoing vitrified-warmed single blastocyst transfer. The live birth rate, birth weight, gestational age, and congenital anomaly rate were within expected ranges and comparable to those observed in an IVF reference population. These findings support the clinical utility and safety of PIEZO-ICSI as a fertilization method in assisted reproductive technology. Future prospective studies with long-term follow-up of offspring are warranted to confirm these findings.

## Supplementary Information

Below is the link to the electronic supplementary material.

Supplementary file1 (DOCX 20 KB)

## Data Availability

The data will be shared with other researchers for further analysis.
