# Supplementary material for: Pregnancy and perinatal outcomes following PIEZO-ICSI with vitrified-warmed single blastocyst transfer: a retrospective cohort study
Source: J Assist Reprod Genet. 2026 May 23;43(7):2267–74. doi: 10.1007/s10815-026-03915-0 (PMC13518730; doi:10.1007/s10815-026-03915-0)
Supplement: Supplementary file 1 [file 10815_2026_3915_MOESM1_ESM.docx]

| **Case No.** | **Group** | **Sex** | **Category** | **Specific anomaly** |
| --- | --- | --- | --- | --- |
| IVF‑01 | IVF | M | Chromosomal | Trisomy 21 |
| IVF‑02 | IVF | M | Gastrointestinal | Duodenal atresia |
| IVF‑03 | IVF | F | Renal/urinary | Bilateral hydronephrosis |
| IVF‑04 | IVF | M | Limb | Left hypodactyly |
| IVF‑05 | IVF | M | Cardiovascular | Persistent left superior vena cava |
| IVF‑06 | IVF | M | Musculoskeletal | Congenital dislocation of left knee |
| IVF‑07 | IVF | M | Craniofacial | Cleft lip and palate |
| IVF‑08 | IVF | F | Craniofacial | Bilateral cleft lip |
| IVF‑09 | IVF | F | Renal/urinary | Congenital hydronephrosis |
| IVF‑10 | IVF | F | Other | Ovarian cyst |
| IVF‑11 | IVF | F | Chromosomal | Trisomy 21 |
| IVF‑12 | IVF | M | Limb | Congenital limb defect |
| IVF‑13 | IVF | F | Chromosomal | Trisomy 18 |
| IVF‑14 | IVF | F | Cardiovascular | Hypoplastic left heart syndrome |
| IVF‑15 | IVF | F | Musculoskeletal | Congenital dislocated hip |
| IVF‑16 | IVF | M | CNS | Holoprosencephaly |
| PIEZO‑01 | PIEZO | F | Cardiovascular | Ventricular septal defect |
| PIEZO‑02 | PIEZO | M | Musculoskeletal | Clubfoot |
| PIEZO‑03 | PIEZO | F | Limb | Polydactyly |
| PIEZO‑04 | PIEZO | M | Renal/urinary | Duplicated renal pelvis |
| PIEZO‑05 | PIEZO | M | Gastrointestinal | Anal atresia |
| PIEZO‑06 | PIEZO | F | Cardiovascular | Ventricular septal defect |
| PIEZO‑07 | PIEZO | M | Craniofacial | Accessory ear |
| PIEZO‑08 | PIEZO | M | Craniofacial | Accessory ear |
| PIEZO‑09 | PIEZO | F | Cardiovascular | Tetralogy of Fallot |
| PIEZO‑10 | PIEZO | F | Renal/urinary | Multicystic dysplastic kidney |
| PIEZO‑11 | PIEZO | M | Limb | Polydactyly |

**Supplementary Table 1.** List of congenital anomalies observed in the study population

M, male; F, female
